# Added Value of Systemic Inflammation Markers in Predicting Clinical Stage T1 Renal Cell Carcinoma Pathologically Upstaged to T3a

**DOI:** 10.3389/fonc.2021.679536

**Published:** 2021-05-31

**Authors:** Hailang Liu, Zhixian Wang, Ejun Peng, Zhiqiang Chen, Kun Tang, Ding Xia

**Affiliations:** Department of Urology, Tongji Hospital, Tongji Medical College, Huazhong University of Science and Technology, Wuhan, China

**Keywords:** renal cell carcinoma, inflammatory markers, nephrometry score, upstaging, pathology

## Abstract

**Objectives:**

We aimed to determine preoperative risk factors associated with pathologic T3a (pT3a) upstaging of clinical T1 (cT1) renal cell carcinomas (RCCs) and develop a novel model capable of accurately identifying those patients at high risk of harboring occult pT3a characteristics.

**Methods:**

A retrospective analysis of 1324 cT1 RCC patients who underwent partial nephrectomy (PN) or radical nephrectomy (RN) was performed. The study cohort was divided into training and testing datasets in a 70:30 ratio for further analysis. Univariable and multivariable logistic regression analyses were performed to identify predictors associated with cT1 to pT3a upstaging and subsequently, those significant risk factors were used to construct models. We used the area under the curve (AUC) to determine the model with the highest discrimination power. Decision curve analyses (DCAs) were applied to evaluate clinical net benefit associated with using the predictive models.

**Results:**

The rates of upstaging were 6.1% (n = 81), 5.8% (n = 54) and 6.8% (n = 27) in the total population, training cohort and validation cohort, respectively. Tumor size, clinical T stage, R.E.N.A.L. (radius, exophytic/endophytic properties, nearness of tumor to collecting system or sinus, anterior/posterior) nephrometry score, lymphocyte to monocyte ratio (LMR), prognostic nutrition index (PNI) and albumin to globulin ratio (AGR) were significantly associated with pT3a upstaging. The model that consisted of R.E.N.A.L. score, LMR, AGR and PNI achieved the highest AUC of 0.70 in the validation cohort and yielded the highest net benefit. In the subpopulation with complete serum lipid profile, the inclusion of low-density lipoprotein cholesterol (LDL-C) and Castelli risk index-I (CRI-I) significantly improved the discrimination of model (AUC = 0.86).

**Conclusions:**

Our finding highlights the importance of systemic inflammation response markers and serum lipid parameters in predicting pT3a upstaging. Our model had relatively good discrimination in predicting occult pT3a disease among patients with cT1 renal lesions, and the use of the model may be greatly beneficial to urologists in risk stratification and management decisions.

## Introduction

Currently, the majority of patients diagnosed with RCC tend to harbor small, organ-confined tumors due to the increased use of abdominal cross-sectional imaging (1). Tailoring individualized, proper surgical management of a localized renal mass involves balancing surgical difficulty and oncologic risk (2). PN has become the standard treatment for clinical stage T1a (cT1a) masses, suggesting its better functional outcomes compared to RN, without compromising cancer control (3, 4). However, among those patients who undergo PN or RN for cT1 masses, up to 4–13% may have occult adverse pathological features, such as perirenal or sinus fat invasion (5–11). Sometimes, it can be challenging for computed tomography to identify microscopic perirenal or sinus fat invasion preoperatively. Thus, when performing PN for cT1 masses, there is a risk of upstaging to pT3a. Tumor upstaging poses a clinical dilemma in refining patient selection for the surgical management of cT1 RCC. Given the increased recurrence risk for pT3a patients after PN, improved understanding of preoperative risk factors would aid in risk stratification and in choosing the best therapeutic approach (7, 10). As a result, it is of great importance to accurately identify those cT1 patients at high risk for pT3a upstaging prior to surgery.

Emerging evidence suggests that systematic inflammation *via* host-tumor interactions is intimately involved in the development and progression of RCC (12–14). Based on this knowledge, systemic inflammatory markers such as neutrophils, lymphocytes, platelets, albumin, C-reactive protein (CRP) and biomarker combination ratios (e.g., neutrophil to lymphocyte ratio [NLR], LMR and platelet to lymphocyte ratio [PLR]) may have potential value in predicting pT3a upstaging in cT1 RCC patients.

In the present study, we sought to determine those predictors independently associated with pathological tumor upstaging, and develop a predictive model capable of precisely identifying cT1 RCC patients at high risk for upstaging.

## Methods

### Patient Selection

**Figure 1:**
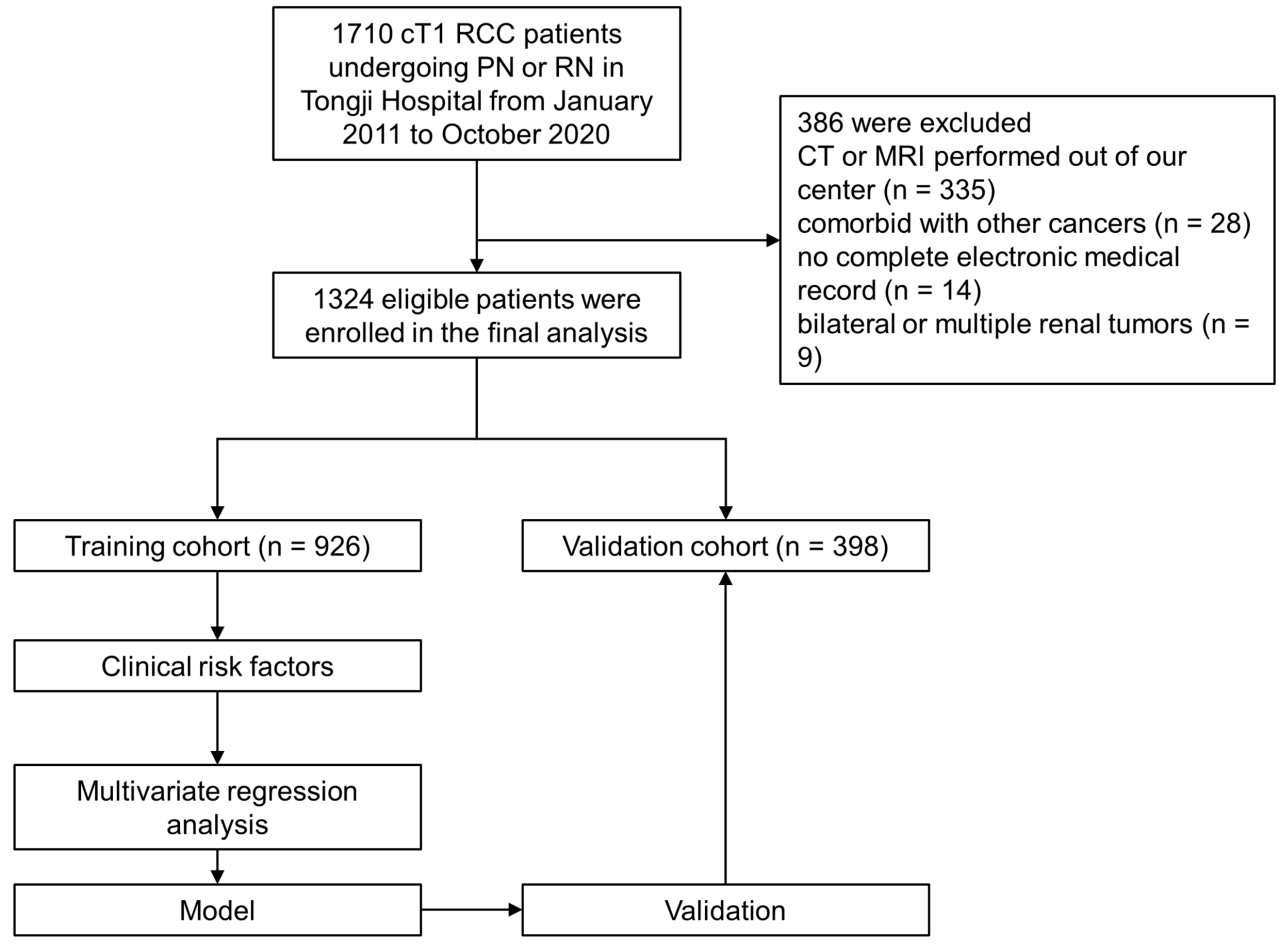
Flow chart of the study.

Following Institutional Review Board approval, clinical and pathologic data of 1,710 RCC patients treated with PN or RN between January 2011–October 2020 at the Tongji Hospital of Tongji Medical College, Huazhong University of Science and Technology were retrospectively collected. The inclusion criteria were as follows: (1) patients aged ≥ 18 years; (2) patients with cT1 (7 cm or less in diameter) lesions with no distant metastasis (15); (3) PN or RN performed for all patients; and (4) complete clinical data, including patient demographics, surgical approach, preoperative laboratory test results, tumor pathology and imaging of the abdomen (computed tomography [CT] or magnetic resonance imaging [MRI]). The exclusion criteria were as follows: (1) patients treated with thermal ablation, neoadjuvant chemotherapy or radiotherapy prior to surgery; (2) patients with comorbid cancers; (3) patients with CT or MRI examinations not performed at our center; (3) patients with bilateral or multiple renal lesions; and (4) patients with incomplete laboratory data. Initially, 1710 patients underwent PN or RN were included, and study flowchart is shown in **Figure 1**.

### Data Collection and Outcomes

We collected baseline demographics, including age, body mass index (BMI), gender, comorbidities, surgical approach, nephrectomy type and tumor characteristics, including size, laterality, pathologic stage, histology, grade and R.E.N.A.L. nephrometry score (16). We also collected preoperative routine laboratory measurements including neutrophil (10^9^/L), lymphocyte (10^9^/L), platelet (10^9^/L), monocyte (10^9^/L), NLR, PLR, LMR, CRP, hemoglobin, albumin, globulin, AGR, PNI, albumin to CRP ratio (ACR), lymphocyte to CRP ratio (LCR), modified Glasgow Prognostic Score (mGPS), total cholesterol (TC), triglyceride (TG), LDL-C, high-density lipoprotein cholesterol (HDL-C), CRI-I and Castelli risk index-II (CRI-I). PNI was calculated as serum albumin (g/L) 5 peripheral blood lymphocyte count (10^9^/L) (17). The mGPS was constructed, using albumin and CRP, as follows: patients with both an elevated CRP (> 10 mg/L) and low albumin (< 35 g/L) were assigned a score of 2; patients in whom only CRP was elevated (> 10 mg/L) were assigned a score of 1 and those with a normal CRP were assigned a score of 0 (18). CRI-I was defined as the TC to HDL-C ratio, while CRI-II was defined as the LDL-C to HDL-C ratio (19). The outcome of our study was upstaging, defined as the presence of T3a RCC at final pathology.

### Statistical Analyses

The primary study cohort was randomly split into training and validation cohorts at a ratio of 70:30. In both the training and validation cohorts, patients were assigned to the pT3a upstaging group and no pT3a upstaging group. Descriptive analyses included the medians and interquartile ranges (IQRs) for continuous variables, and frequencies and proportions were reported for categorical variables. In the analysis of tumor upstaging, categorical variables were compared with Pearson χ^2^ test or Fisher’s exact test, and continuous variables were compared with Mann-Whitney U test in both the training and validation cohorts. For the development of predictive models, univariable logistic regression analyses were first performed to determine the variables associated with pT3a upstaging. If a variable was significantly associated with upstaging at the p < 0.05 level, then it was included in the multivariate logistic regression analyses. On multivariate logistic regression analysis, those variables at the p < 0.05 level were considered to be statistically independently associated with pT3a upstaging. To overcome the problem of multicollinearity among tumor size, clinical T stage, R.E.N.A.L. score and R.E.N.A.L. complexity (low 4–6, moderate 7–9, high 10–12), we then developed four different predictive models based on those clinically relevant and significant predictors. Given the important role of serum lipid levels in predicting prognosis of RCC, we further investigated clinical implications of serum lipid levels and its combination ratios in predicting pT3a upstaging in subgroup of patients with complete preoperative serum lipid profile (20–22). The discrimination accuracy of these models in our cohort was evaluated using the receiver operating characteristic (ROC)-derived AUC. Comparisons between ROC curves were performed using the method described by DeLong et al. (23). DCA was performed to determine the clinical net benefit associated with using the predictive models at different threshold probabilities in the patient cohort.

Data were analyzed using the statistical software SPSS version 24.0 (IBM Corp., NY, USA) and R software (Version 3.6.0; https://www.R-project.org). All tests were two- sided, with a significance level set at p < 0.05.

## Results

### Characteristics of the Study Cohort

**Figure 2:**
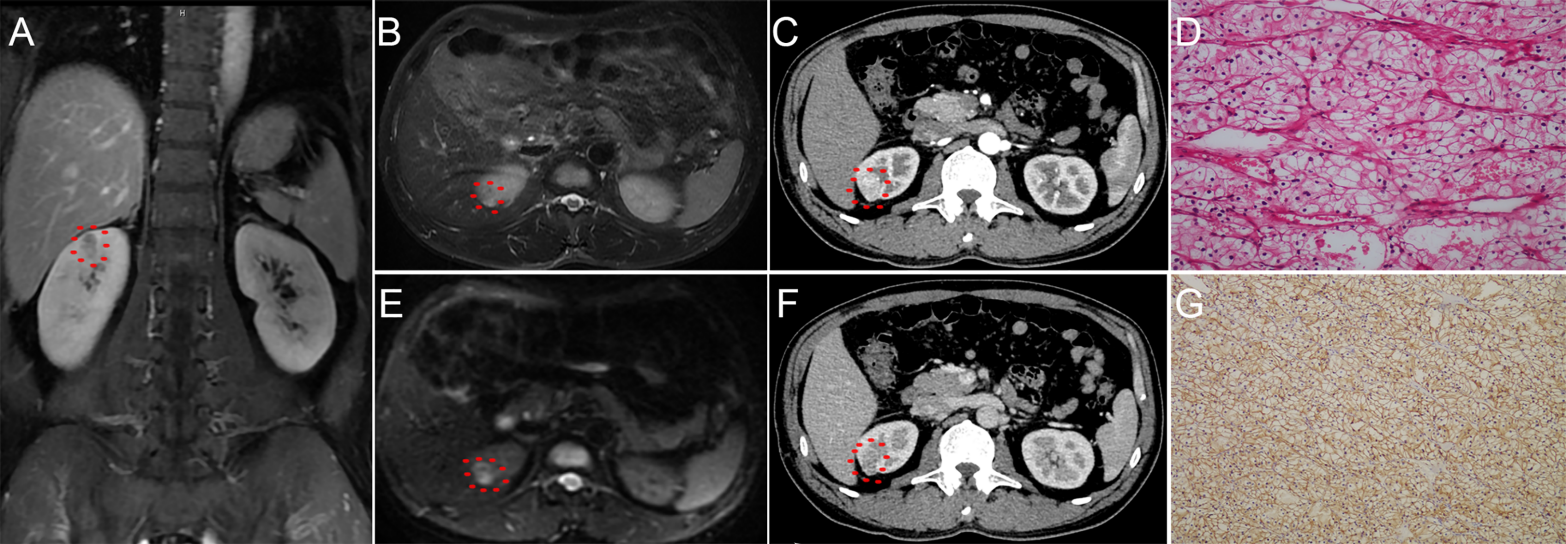
Representative radio-pathological matching case of a clinical T1 renal lesion pathologically upstaged to T3a. Patient summary: a 46-year-old man with a right clinical T1 renal lesion; BMI 25.3 kg/m2; preoperative C-reactive protein 0.2 mg/L; TC 4.37 mmol/L; TG 1.05 mmol/L; HDL-C 1.25 mmol/L; LDL-C 2.86 mmol/L; CRI-I 3.5; CRI-II 2.3; NLR 2.1; PLR 168.9; LMR 3.7; AGR 1.6; PNI 49.5; lesion dimension on MRI 1.6 cm; R.E.N.A.L. score 7; final pathology: clear cell renal cell carcinoma, Fuhrman grade I, sinus fat invasion. **(A)** coronal perfusion-weighted MRI; **(B)** axial T2-weighted MRI; **(C, F)** dynamic contrast-enhanced CT imaging; **(D)** representative pathology of partial nephrectomy specimen; **(E)** axial diffusion-weighted MRI with b value set at 600 s/mm2; **(G)** immunohistochemical staining figure regarding the positive expression of carbonic anhydrase IX.

Strictly conforming to the inclusion and exclusion criteria, 1,324 cT1 RCC patients who underwent PN or RN between January 2011– October 2020 at our center were enrolled and randomly split into the training (n = 926) and validation (n = 398) cohorts (**Figure 1**). Demographic and clinical characteristics of total population, training and validation cohorts are shown in **Tables 1** and **2**, respectively. The tumor lesion imaging and pathologic characteristics are presented in **Tables 3** and **4**. Of the patients with cT1 disease, 81 were upstaged to pT3a at final pathology in the entire cohort, while the rate of upstaging was 5.8% and 6.8% in the training and validation cohorts, respectively. Pathologically-upstaged tumors had greater size (5.0 vs. 3.8 cm, p < 0.001), higher R.E.N.A.L. scores (9 vs. 7, p < 0.001), higher grades (32.1% vs. 8.5% grade III–IV, p < 0.001) and lower incidences of clear cell histology (66.7% vs. 79.6%, p < 0.001). Preoperative laboratory test results showed that patients in the non-pT3a group had higher neutrophil counts (3.98 vs. 3.2410^9^/L, p < 0.001), higher platelet counts (226 vs. 20510^9^/L, p = 0.003), higher monocyte counts (0.53 vs. 0.4510^9^/L, p = 0.001), lower lymphocyte counts (1.58 vs. 1.7510^9^/L, p = 0.023) and elevated globulin (29.5 vs. 27.3 g/L, p < 0.001). In terms of biomarker combination ratios, levels of NLR, PLR, LMR, AGR and PNI were significantly different in the non-pT3a upstaging group, compared with the pT3a upstaging group (all p < 0.05). Representative case illustrating presence of pT3a upstaging at final pathology was shown in **Figure 2**. All baseline characteristics were comparable between the training and validation cohorts. Patients with complete preoperative serum lipid profile were limited to 601, including 553 non-upstaged and 48 upstaged patients. Demographic, clinical and tumor characteristics are summarized in **Supplementary Tables 1** and **2**. Among these individuals, upstaged patients had greater tumor size (5.0 vs. 3.9 cm, p = 0.002), higher R.E.N.A.L. scores (9 vs. 8, p = 0.009), higher grades (29.2% vs. 8.0% grade III–IV, p < 0.001), elevated neutrophil counts (3.65 vs. 3.2010^9^/L, p = 0.02), elevated platelet counts (231 vs. 20810^9^/L, p = 0.009), decreased HDL-C (0.91 vs. 1.02 mmol/L, p = 0.001) and elevated LDL-C (2.61 vs. 2.57 mmol/L, p = 0.001). Additionally, with regard to biomarker combination ratios, levels of NLR, PLR, LMR, AGR, LCR, CRI-I and CRI-II significantly differed between the non-pT3a upstaging and pT3a upstaging groups, and high mGPS occurred more frequently in the pT3a upstaging group (all p < 0.05).

**Table 1:** Baseline demographic and clinical characteristics of study cohort.

	No pT3a Upstaging	pT3a Upstaging	p value
Patients (%)	1243 (93.9)	81 (6.1)	
Patient characteristics			
Age (years), median [IQR]	53 (46-61)	53 (46-62)	0.842
Gender, N (%)			0.921
Male	822 (66.1)	54 (66.7)	
Female	421 (33.9)	27 (33.3)	
BMI (kg/m^2^), median [IQR]	24.2 (22.0-26.4)	24.1 (22-25.8)	0.675
Comorbidities, N (%)			
Hypertension	367 (29.5)	20 (24.7)	0.354
Diabetes mellitus	138 (11.1)	10 (12.3)	0.731
CAD/CVD	72 (5.8)	3 (3.7)	0.619
Surgical approach, N (%)			0.577
Laparoscopic	1156 (93.0)	74 (91.4)	
Open	87 (7.0)	7 (8.6)	
Nephrectomy type, N (%)			<0.001
PN	799 (64.3)	29 (35.8)	
RN	444 (35.7)	52 (64.2)	
ASA score, N (%)			0.834
1-2	1114 (89.6)	72 (88.9)	
3-4	129 (10.4)	9 (11.1)	
Preoperative laboratory examination			
Neutrophil count (10^9/L), median [IQR]	3.24 (2.62-4.12)	3.98 (3.14-5.09)	<0.001
Lymphocyte count (10^9/L), median [IQR]	1.75 (1.42-2.15)	1.58 (1.39-1.86)	0.023
Platelet count (10^9/L), median [IQR]	205 (171-247)	226 (189-278)	0.003
Monocyte count (10^9/L), median [IQR]	0.45 (0.36-0.56)	0.53 (0.39-0.69)	0.001
NLR, median [IQR]	1.9 (1.4-2.5)	2.3 (1.7-3.5)	<0.001
PLR, median [IQR]	117.2 (91.1-146.3)	142.5 (104.9-179.2)	<0.001
LMR, median [IQR]	3.9 (3.1-5.1)	3.2 (2.5-4.1)	<0.001
Hemoglobin (g/L), median [IQR]	136 (124-147)	132 (115-145)	0.047
Albumin (g/L), median [IQR]	41.0 (38.8-43.1)	40.2 (37.8-42.6)	0.087
Globulin (g/L), median [IQR]	27.3 (24.9-30.3)	29.5 (25.5-33.9)	<0.001
AGR, median [IQR]	1.5 (1.3-1.7)	1.4 (1.1-1.6)	<0.001
PNI, median [IQR]	49.9 (46.8-53.3)	48.4 (45.6-51.5)	0.007
Serum creatinine (µmol/L), median [IQR]	74 (62-87)	83 (66-96)	0.004
TC^†^ (mmol/L), median [IQR]	4.07 (3.57-4.59)	3.98 (3.37-4.39)	0.118

AGR, albumin to globulin ratio; ASA, American Society of Anesthesia; BMI, body mass index; CAD, coronary arterial disease; CVD, cerebrovascular disease; IQR, interquartile range; LMR, lymphocyte to monocyte ratio; NLR, neutrophil to lymphocyte ratio; PLR, platelet to lymphocyte ratio; PN, partial nephrectomy; PNI, prognostic nutrition index; RN, radical nephrectomy; TC, total cholesterol.

^†^Limited to the 1288 patients, including 1207 non-upstaged and 81upstaged patients.

**Table 2:** Tumor characteristics between those with and without pT3a renal cell carcinoma in study cohort.

	No pT3a Upstaging	pT3a Upstaging	p value
Patients, (%)	1243 (93.9)	81 (6.1)	
Laterality, N (%)			0.461
Left	605 (48.7)	36 (44.4)	
Right	638 (51.3)	45 (55.6)	
Hilar location, N (%)			0.320
No	1063 (85.5)	66 (81.5)	
Yes	180 (14.5)	15 (18.5)	
Tumor location, N (%)			0.603
Posterior	474 (38.1)	32 (39.5)	
Anterior	478 (38.5)	27 (33.3)	
Lateral	291 (23.4)	22 (27.2)	
Tumor size (cm), median [IQR]	3.8 (2.9-5.0)	5.0 (3.5-6.2)	<0.001
Clinical T stage, N (%)			0.001
T1a	682 (54.9)	29 (35.8)	
T1b	561 (45.1)	52 (64.2)	
R.E.N.A.L. score, median [IQR]	7 (6-9)	9 (7-9)	<0.001
R.E.N.A.L. complexity, N (%)			0.002
Low [4–6]	414 (33.3)	14 (17.3)	
Moderate [7–9]	659 (53.0)	47 (58.0)	
High [10–12]	170 (13.7)	20 (24.7)	
Tumor histology, N (%)			0.010
Clear cell RCC	990 (79.6)	54 (66.7)	
Papillary RCC	101 (8.1)	10 (12.3)	
Chromophobe RCC	74 (6.0)	5 (6.2)	
Other (Benign, Mixed, Cystic, etc.)	78 (6.3)	12 (14.8)	
Histologic grade, N (%)			<0.001
Low (I–II)	960 (77.2)	36 (44.4)	
High (III–IV)	106 (8.5)	26 (32.1)	
Unknown or n/a	177 (14.3)	19 (23.5)	

IQR, interquartile range; n/a, not applicable; RCC, renal cell carcinoma; R.E.N.A.L. nephrometry score consists of [R]adius [tumor size as maximal diameter], [E]xophytic/endophytic properties of the tumor, [N]earness of tumor deepest portion to the collecting system or sinus, [A]nterior [a]/posterior [p] descriptor and the [L]ocation relative to the polar line.

**Table 3:** Baseline demographic and clinical characteristics of training and validation cohort.

	Training cohort			Validation cohort		
	No pT3a Upstaging	pT3a Upstaging	p value	No pT3a Upstaging	pT3a Upstaging	p value
Patients (%)	872 (94.2)	54 (5.8)		371 (93.2)	27 (6.8)	
Patient characteristics						
Age (years), median [IQR]	54 (46-61)	53 (44-62)	0.748	53 (47-61)	54 (50-62)	0.408
Gender, N (%)			0.648			0.616
Male	571 (65.5)	37 (68.5)		251 (67.7)	17 (63.0)	
Female	301 (34.5)	17 (31.5)		120 (32.3)	10 (37.0)	
BMI (kg/m^2^), median [IQR]	24.1 (22.0-26.1)	23.9 (21.9-25.3)	0.348	24.2 (22.1-26.7)	25.2 (22.7-26.4)	0.624
Comorbidities, N (%)						
Hypertension	243 (27.9)	13 (24.1)	0.545	124 (33.4)	7 (25.9)	0.423
Diabetes mellitus	99 (11.4)	5 (9.3)	0.636	39 (10.5)	5 (18.5)	0.203
CAD/CVD	50 (5.7)	1 (1.9)	0.356	22 (5.9)	2 (7.4)	0.673
Surgical approach, N (%)			0.568			0.729
Laparoscopic	819 (93.9)	50 (92.6)		337 (90.8)	24 (88.9)	
Open	53 (6.1)	4 (7.4)		34 (9.2)	3 (11.1)	
Nephrectomy type, N (%)			<0.001			0.005
PN	550 (63.1)	18 (33.3)		249 (67.1)	11 (40.7)	
RN	322 (36.9)	36 (66.7)		122 (32.9)	16 (59.3)	
ASA score, N (%)			0.269			0.338
1-2	784 (89.9)	46 (85.2)		330 (88.9)	26 (96.3)	
3-4	88 (10.1)	8 (14.8)		41 (11.1)	1 (3.7)	
Preoperative laboratory examination						
Neutrophil count (10^9/L), median [IQR]	3.25 (2.64-4.11)	3.95 (3.15-5.14)	<0.001	3.20 (2.53-4.21)	4.01 (3.27-5.04)	0.011
Lymphocyte count (10^9/L), median [IQR]	1.74 (1.42-2.12)	1.57 (1.34-1.83)	0.032	1.77 (1.42-2.22)	1.59 (1.43-1.90)	0.348
Platelet count (10^9/L), median [IQR]	204 (170-245)	227 (194-283)	0.003	207 (177-248)	214 (185-264)	0.334
Monocyte count (10^9/L), median [IQR]	0.45 (0.36-0.56)	0.54 (0.39-0.71)	0.005	0.45 (0.36-0.56)	0.48 (0.41-0.65)	0.105
NLR, median [IQR]	1.9 (1.4-2.5)	2.4 (2.0-3.6)	<0.001	1.8 (1.4-2.5)	2.1 (1.7-2.9)	0.046
PLR, median [IQR]	119.4 (92.1-146.9)	148.7 (103.9-192.2)	<0.001	113.5 (89.2-143.6)	115.3 (107.1-173.6)	0.191
LMR, median [IQR]	3.9 (3.1-5.1)	2.9 (2.3-3.9)	<0.001	4.0 (3.2-5.2)	3.8 (2.9-4.3)	0.127
Hemoglobin (g/L), median [IQR]	136 (124-147)	131 (109-145)	0.039	138 (125-148)	138 (119-147)	0.607
Albumin (g/L), median [IQR]	40.9 (38.7-43.1)	40.0 (37.9-42.5)	0.219	41.2 (39.3-43.2)	40.5 (37.5-43.0)	0.227
Globulin (g/L), median [IQR]	27.4 (24.9-30.4)	30.2 (25.6-35.9)	0.001	27.2 (24.9-29.8)	28.8 (25.5-31.3)	0.096
AGR, median [IQR]	1.5 (1.3-1.7)	1.4 (1.1-1.6)	0.002	1.5 (1.4-1.7)	1.4 (1.3-1.6)	0.071
PNI, median [IQR]	49.7 (46.8-53.2)	48.3 (45.5-51.6)	0.025	50.2 (46.7-54.0)	48.7 (46.3-50.7)	0.106
Serum creatinine (µmol/L), median [IQR]	74 (62-87)	88 (67-98)	0.004	74 (62-87)	78 (64-92)	0.372

AGR, albumin to globulin ratio; ASA, American Society of Anesthesia; BMI, body mass index; CAD, coronary arterial disease; CVD, cerebrovascular disease; IQR, interquartile range; LMR, lymphocyte to monocyte ratio; NLR, neutrophil to lymphocyte ratio; PLR, platelet to lymphocyte ratio; PN, partial nephrectomy; PNI, prognostic nutrition index; RN, radical nephrectomy.

**Table 4:** Tumor characteristics between those with and without pT3a renal cell carcinoma in training and validation cohort.

	Training cohort			Validation cohort		
	No pT3a Upstaging	pT3a Upstaging	p value	No pT3a Upstaging	pT3a Upstaging	p value
Patients, (%)	872 (94.2)	54 (5.8)		371 (93.2)	27 (6.8)	
Laterality, N (%)			0.957			0.205
Left	407 (46.7)	25 (46.3)		198 (53.4)	11 (40.7)	
Right	465 (53.3)	29 (53.7)		173 (46.6)	16 (59.3)	
Hilar location, N (%)			0.302			0.767
No	740 (84.9)	43 (79.6)		323 (87.1)	23 (85.2)	
Yes	132 (15.1)	11 (20.4)		48 (12.9)	4 (14.8)	
Tumor location, N (%)			0.506			0.901
Posterior	339 (38.9)	21 (38.9)		135 (36.4)	11 (40.7)	
Anterior	329 (37.8)	17 (31.5)		149 (40.2)	10 (37.0)	
Lateral	204 (23.3)	16 (29.6)		87 (23.4)	6 (22.3)	
Tumor size (cm), median [IQR]	3.8 (2.8-5.0)	5.2 (3.5-6.4)	<0.001	3.9 (2.9-5.0)	4.5 (3.7-6.0)	0.017
Clinical T stage, N (%)			0.004			0.095
T1a	483 (55.4)	19 (35.2)		199 (53.6)	10 (37.0)	
T1b	389 (44.6)	35 (64.8)		172 (46.4)	17 (63.0)	
R.E.N.A.L. score, median [IQR]	8 (6-9)	9 (7-9)	0.001	7 (5-9)	9 (7-10)	0.010
R.E.N.A.L. complexity, N (%)			0.039			0.026
Low [4–6]	269 (30.8)	10 (18.5)		145 (39.1)	4 (14.8)	
Moderate [7–9]	484 (55.5)	31 (57.4)		175 (47.2)	16 (59.3)	
High [10–12]	119 (13.7)	13 (24.1)		51 (13.7)	7 (25.9)	
Tumor histology, N (%)			0.001			0.998
Clear cell RCC	691 (79.2)	31 (57.4)		299 (80.6)	23 (85.2)	
Papillary RCC	75 (8.6)	9 (16.7)		26 (7.0)	1 (3.7)	
Chromophobe RCC	48 (5.5)	3 (5.6)		26 (7.0)	2 (7.4)	
Other (Benign, Mixed, Cystic, etc.)	58 (6.7)	11 (20.3)		20 (5.4)	1 (3.7)	
Histologic grade, N (%)			<0.001			0.001
Low (I–II)	673 (77.2)	22 (40.7)		287 (77.4)	14 (51.9)	
High (III–IV)	75 (8.6)	17 (31.5)		31 (8.4)	9 (33.3)	
Unknown or n/a*	124 (14.2)	15 (27.8)		53 (14.2)	4 (14.8)	

IQR, interquartile range; n/a, not applicable; RCC, renal cell carcinoma; R.E.N.A.L. nephrometry score consists of [R]adius [tumor size as maximal diameter], [E]xophytic/endophytic properties of the tumor, [N]earness of tumor deepest portion to the collecting system or sinus, [A]nterior [a]/posterior [p] descriptor and the [L]ocation relative to the polar line.

### Univariable and Multivariable Analysis

**Figure 3:**
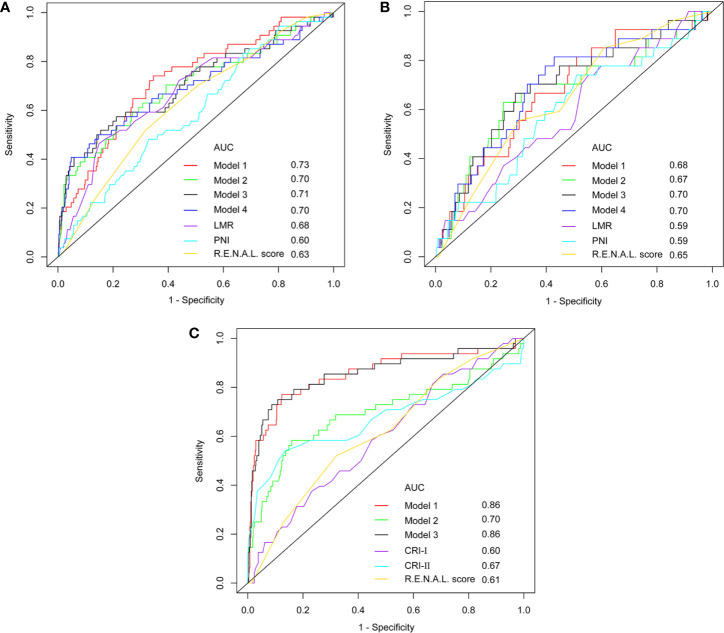
The ROC curve analyses for models in training cohort **(A)**, validation cohort **(B)** and subpopulation **(C)**.

At univariable analyses, neutrophil, lymphocyte, platelet, monocyte, NLR, PLR, LMR, globulin, AGR, PNI, tumor size, clinical T stage, R.E.N.A.L. score and R.E.N.A.L. complexity represented predictors of pT3a upstaging (all p < 0.05). At multivariable analyses, LMR, PNI, AGR, tumor size, clinical T stage and R.E.N.A.L. score were significantly associated with pT3a upstaging, as displayed in **Table 5** (all p < 0.05). To avoid the collinearity of R.E.N.A.L. score with tumor size and clinical T stage, these independent predictors were fitted in four different multivariable models. Model 3 achieved an AUC of 0.71 and 0.70 in the training and validation cohort, respectively (**Figures 3A, B**). It represented the basis for the novel model predicting pT3a upstaging. A corresponding nomogram regarding calculating individualized risk of pT3a upstaging for each patient was subsequently constructed based on model 3 (**Supplementary Figure 1**). In the model 3, decreased LMR (odds ratio [OR] = 0.72; 95% confidence interval [CI] = 0.55–0.93; p = 0.011], decreased AGR (OR = 0.21; 95% CI = 0.06–0.71; p = 0.012), increased PNI (OR = 1.10; 95% CI = 1.01–1.18; p = 0.024) and increased R.E.N.A.L. score (OR = 1.26; 95% CI = 1.06–1.48; p = 0.008) increased the likelihood of tumor upstaging. To investigate the role of serum lipid in predicting upstaging, a subset of cT1 RCC patients with complete preoperative serum lipid profile was analyzed. In univariable analysis, NLR, PLR, hemoglobin, AGR, tumor size, CRP, mGPS, R.E.N.A.L. score, HDL-C, LDL-C, CRI-I and CRI-II were associated with a higher risk of pT3a upstaging (all p < 0.05) (**Supplementary Table 3**). At multivariable analysis, R.E.N.A.L. score, tumor size, LDL-C, CRI-I and CRI-II were statistically significant predictors of pT3a upstaging. Model 1 achieved the highest AUC of 0.86 (**Figure 3C**). In model 1, increased R.E.N.A.L. score (OR = 1.27; 95% CI = 1.03–1.56; p = 0.028), decreased LDL-C (OR = 0.13; 95% CI = 0.07–0.23; p < 0.001) and increased CRI-I (OR = 2.19; 95% CI = 1.55–3.09; p < 0.001) were independently associated with an increased risk of tumor upstaging.

**Table 5:** Multivariable logistic regression analyses predicting pathologic T3a upstaging in patients diagnosed with clinical T1 renal masses undergoing nephrectomy.

	Model 1		Model 2		Model 3		Model 4	
	OR (95% CI)	p value	OR (95% CI)	p value	OR (95% CI)	p value	OR (95% CI)	p value
NLR	1.04 (0.87-1.23)	0.691	1.03 (0.87-1.22)	0.719	1.02 (0.87-1.20)	0.786	1.02 (0.87-1.20)	0.811
PLR	1.00 (0.99-1.01)	0.150	1.00 (0.99-1.01)	0.106	1.00 (0.99-1.01)	0.095	1.00 (0.99-1.01)	0.085
LMR	0.71 (0.54-0.93)	0.011	0.71 (0.55-0.92)	0.011	0.72 (0.55-0.93)	0.011	0.72 (0.55-0.93)	0.012
Hemoglobin	0.99 (0.97-1.001)	0.063	0.99 (0.97-1.001)	0.08	0.99 (0.97-1.003)	0.123	0.99 (0.97-1.003)	0.101
AGR	0.30 (0.09-1.02)	0.053	0.25 (0.07-0.86)	0.027	0.21 (0.06-0.71)	0.012	0.21 (0.06-0.73)	0.013
PNI	1.09 (1.01-1.19)	0.028	1.10 (1.01-1.18)	0.024	1.10 (1.01-1.18)	0.024	1.10 (1.01-1.18)	0.023
Tumor size	1.43 (1.18-1.75)	<0.001	–	–	–	–	–	–
Clinical T stage								
T1a	–		1(Ref.)		–		–	
T1b	–	–	1.94 (1.07-3.52)	0.028	–	–	–	–
R.E.N.A.L. score	–	–	–	–	1.26 (1.06-1.48)	0.008	–	–
R.E.N.A.L. complexity								
Low [4–6]	–		–		–		1(Ref.)	
Moderate [7–9]	–	–	–	–	–	–	1.67 (0.80-3.52)	0.176
High [10–12]	–	–	–	–	–	–	2.61 (1.08-6.32)	0.034

AGR, albumin to globulin ratio; CI, confidence interval; LMR, lymphocyte to monocyte ratio; NLR, neutrophil to lymphocyte ratio; OR, odds ratio; PLR, platelet to lymphocyte ratio; PNI, prognostic nutrition index; Ref, reference.

### Performance of Models

**Figure 4:**
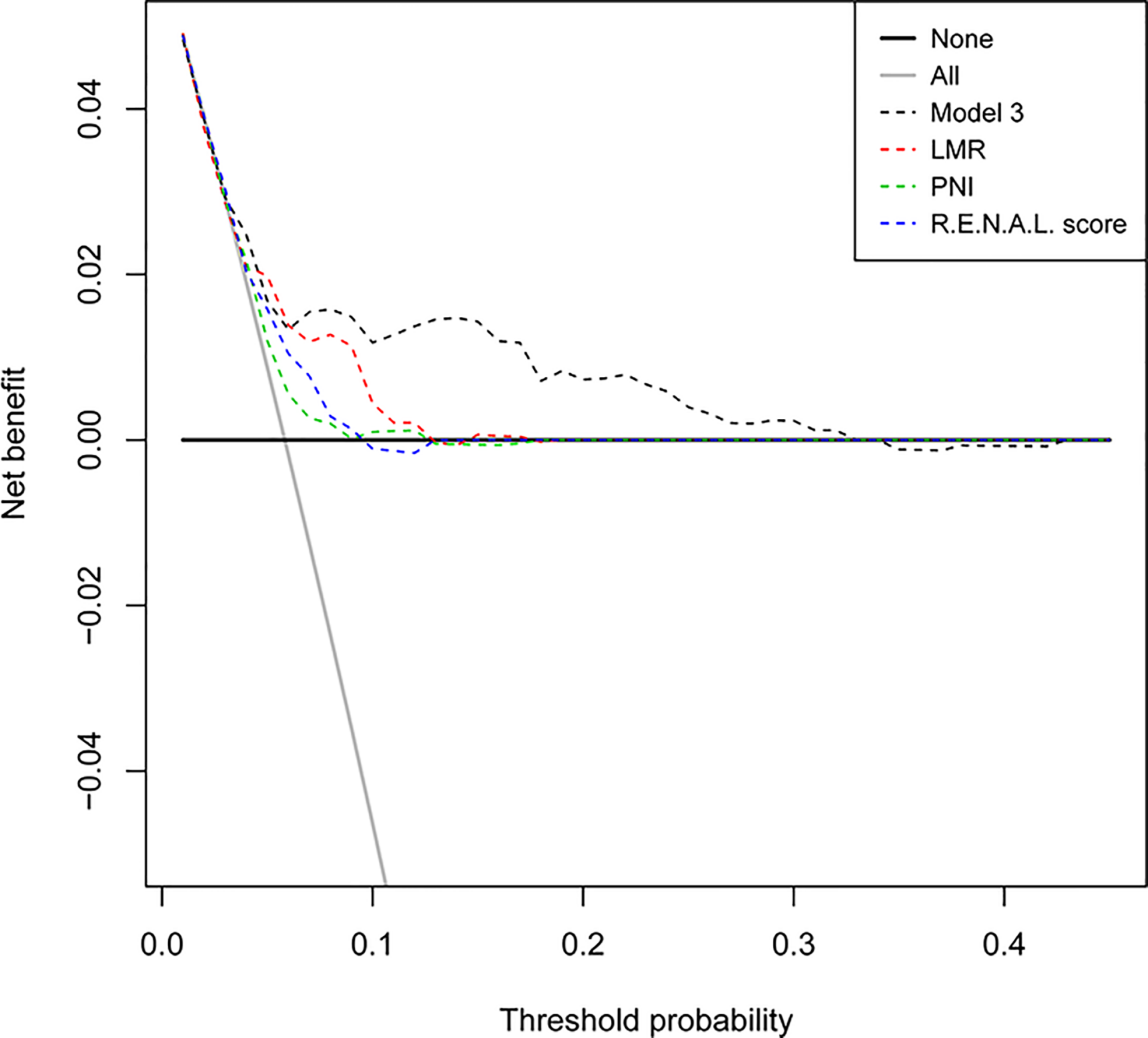
Decision-curve analyses demonstrating the net benefit associated with the use of the models for the prediction of upstaging.

Among these models, 1 and 3 yielded the highest AUCs of 0.73 and 0.71, respectively (**Figure 3A**). Nonetheless, in the validation cohort, model 3 and 4 yielded the highest AUCs of 0.70 and 0.70, respectively (**Figure 3B**). Using the DeLong method with Bonferroni correction, the AUC of the models were significantly greater than those of single variables. Accordingly, model 3 was considered to be reasonably well, relative to other predictive models. In addition, in the subgroup of patients with complete serum lipid profiles, both model 1 and model 3 achieved the highest AUC of 0.86 (**Figure 3C**). This result indicated that the levels of serum lipid might play a more important role than other biomarkers in predicting tumor upstaging. In **Figure 4**, which depicted the prediction of pT3a upstaging, the use of model 3 resulted in the highest net benefit, as compared with other single clinical parameters.

## Discussion

Over the last two decades, the incidence of cT1 renal lesions has increased dramatically, largely due to the wide use of abdominal cross-sectional imaging (1, 24). Most patients diagnosed with cT1 RCC eventually undergo extirpative surgery; however, 4–13% of these masses are found to have occult adverse pathological features at final pathology, such as perirenal or sinus fat invasion, and thus should be diagnosed as pT3a RCC (5–11). Currently, for the management of cT1 RCC, PN is considered a standard of care in clinical practice guidelines, and there has been a continuous increase in the utilization of PN over RN (24). It has been demonstrated that RCC patients upstaged to pT3a after surgical excision seem to have a worse oncological outcome than those non-upstaged patients (7, 25, 26). Moreover, among the upstaged patients, those undergoing PN seem to have inferior recurrence-free survival relative to those undergoing RN (10, 11). There exists a risk of tumor upstaging that can potentially jeopardize the survival of upstaged patients undergoing PN. Therefore, accurately identifying those cT1 RCC patients preoperatively who are most likely to be upstaged is extremely important, and this may help clinicians in decision-making and counseling patents. The present study was undertaken to accomplish the development and validation of the models for prediction of upstaging.

In our study, the rate of upstaging was 6.1%, consistent with the 4–13% rate reported in previous literature (5–11). Pathologically upstaged tumors had greater size, higher R.E.N.A.L. scores, higher grades and lower incidences of clear cell histology, also appreciated by Veccia et al. (11, 27) and Hamilton et al. (28) in their respective study series. Performing PN on tumors was more frequent in the non-pT3a upstaging group, and this may be attributed to the fact that complex lesions were mainly managed by RN. There were significant differences between the groups with respect to laboratory test results and biomarker composite ratios. When patients were limited to those with complete serum lipid profiles, upstaged patients had greater tumor size, higher R.E.N.A.L. scores, higher grades, elevated neutrophil counts, elevated platelet counts, decreased HDL-C and elevated LDL-C. There was no difference between the non- and upstaged groups with regard to other clinical parameters including age, gender, BMI, comorbidities, lymphocyte counts, monocyte counts, albumin, globulin, CRP, ACR, PNI, TC and TG. In contrast to our observations, Fukui et al. (29) reported that level of CRP in the upstaged group was higher than that of the non-upstaged group.

On separate multivariable analyses, LMR, PNI, AGR, tumor size, clinical T stage and R.E.N.A.L. score were independent predictors of pT3a upstaging. Similarly, tumor size and R.E.N.A.L. score was linked to pT3a upstaging by several studies (5, 6, 11, 27, 29). Interestingly, Correa et al. (30) observed that higher R.E.N.A.L. score and tumor diameter were associated with tumor malignancy and grading. Moreover, a recent analysis demonstrated that RCCs with higher R.E.N.A.L. scores had higher Ki67 expression, a widely used marker of cell proliferation (31). Cumulatively, this evidence indirectly supported our conclusion. Veccia et al. (27) found that increased age was significantly associated with pT3a upstaging. Jeong et al. (32) also reported that the risk of pT3a upstaging was found to be associated with age. This finding was not consistent with our study or other previous publications (6, 11, 29), indicating that age was not a robust and consistent predictor of tumor upstaging. Noteworthy in the study by Gorin et al. (6) was that hilar location became significant as an independent predictor of tumor upstaging. In contrast, we failed to find the association between hilar location and pT3a upstaging. This might partly be due to the fact that the rate of hilar-located tumors in the analysis by Gorin et al. (6) was relatively high (i.e., 46%).

It has been confirmed that systemic inflammatory response is associated with survival in patients with RCC (12–14). The systemic inflammatory markers mainly involved three categories of indices: differential blood cell counts (i.e., monocytes, lymphocytes, platelets and neutrophils), concentration of specific serum proteins (i.e., CRP and albumin) and combinations of these indices (i.e., NLR, PLR, LMR and mGPS) (33). Albisinni et al. (34) reported that localized RCC patients with elevated NLR were more likely to present with ≥ pT3 stage. As noted above, it appears apparent that laboratory test results may be related to the presence of pT3a upstaging. As a result, LMR, AGR and PNI were found to be associated with upstaging on separate multivariable analyses. In addition, studies have demonstrated that lipid metabolism disorders play an important role in carcinogenesis and development (35). Therefore, we further analyzed the association between serum lipid and pT3a upstaging in the subpopulation. Not surprisingly, we found that HDL-C, LDL-C, CRI-I and CRI-II were independent predictors of upstaging, whereas TC and TG did not. Given the aforementioned results, our analyses suggested that systemic inflammatory markers have great value in predicting occult pT3a disease.

Counseling cT1 RCC patients on their risk of pT3a upstaging based on preoperative clinical parameters and peripheral blood-derived systemic inflammatory response markers seems logical. We included those independent predictors of pT3a upstaging to construct predictive models using multivariate logistic regression analysis. Model 3, which consisted of LMR, AGR, PNI and R.E.N.A.L. score, outperformed other models in predicting occult pT3a disease in both the training and validation cohorts. In a comparable study by Carlos et al. (36), the AUC of the model predicting stage pT3a was 0.86, which was higher than that reported in our study (0.86 vs. 0.71). Besides, the model developed by Nocera et al. (37) also appeared to outperform our model (0.81 vs. 0.71). Discrepancy in performance of predictive models between our and the former two studies may be attributed to the fact that there were more participants enrolled in our series, and the rate of upstaging in our patient cohort was significantly lower. However, it should be noted that the performance of predictive model derived from data in the subpopulation (AUC = 0.86) was not inferior to that of Carlos et al. (36) or Nocera et al. (37). Considering the small sample size in the subpopulation, the true predictive value of serum lipid parameters should be further, externally validated in large datasets. Thus, we did not recommend inclusion of the lipid profile parameters into the final proposed model. Our results implied that systemic inflammatory response markers play a pivotal role in the construction of predictive models.

Our novel model is based on clinical parameters and laboratory test results. As systemic inflammatory response markers are easily measurable and inexpensive in the clinical setting, it would be convenient for clinicians to calculate the risk of harboring occult pT3a disease for cT1 RCC patients preoperatively. From the patients’ perspectives, the use of our model may influence their treatment decision-making and provide them realistic expectations regarding their prognoses. Our model may be most useful for those cT1 patients who embark on active surveillance or ablation therapy, for which the pendulum may shift toward surgery, and for those cT1 patients who embark on PN, for which the pendulum may swing toward RN.

Despite several strengths, the present study is not without limitations. Firstly, due to the inclusion of patients entirely from a single center, the retrospective nature of the current study makes it subject to a selection bias. Our conclusions may not be applicable to other hospital settings. Secondly, our analysis regarding upstaging was limited by the small sample size of 81 pT3a RCCs. Undoubtedly, this small number of upstaged lesions restricted our ability to examine differences between the groups. Of note, some pathologic characteristics (i.e., sinus fat invasion and renal vein and muscular branch invasion) were not typically described by pathologists in our center until a few years ago, potentially resulting in an underestimation of the pT3 incidence. Thirdly, since both PN and RN patients were included in our study, variability between the nephrectomy types may have confounded the rates of tumor upstaging.

## Conclusions

In conclusion, we have demonstrated that systemic inflammation response makers could provide valuable adjunct information to gauge risk of T3a disease and guide treatment decisions among cT1 RCC patients. Considering current limitations in differentiating truly localized RCCs from those at high risk of harboring occult pT3a disease, our model may be a good risk stratification tool. Of course, further validation in multiple institutions with large sample sizes is warranted.

## Data Availability Statement

The raw data supporting the conclusions of this article will be made available by the authors, without undue reservation.

## Ethics Statement

Written informed consent was obtained from the individual(s) for the publication of any potentially identifiable images or data included in this article.

## Author Contributions

HL and DX designed the research. ZW, HL, EP, and ZC collected, analyzed, and interpreted the clinical data. HL and ZW wrote and revised the manuscript. KT and DX revised the manuscript. All authors contributed to the article and approved the submitted version.

## Conflict of Interest

The authors declare that the research was conducted in the absence of any commercial or financial relationships that could be construed as a potential conflict of interest.

## Supplementary Material

Supplementary Figure 1 Nomogram for predicting probability of pT3a upstaging for newly diagnosed cT1 RCC patients.
