# Supplementary figures and images for: Added Value of Systemic Inflammation Markers in Predicting Clinical Stage T1 Renal Cell Carcinoma Pathologically Upstaged to T3a
Source: Front Oncol. 2021 May 31;11:679536. doi: 10.3389/fonc.2021.679536 (PMC8202414; doi:10.3389/fonc.2021.679536)

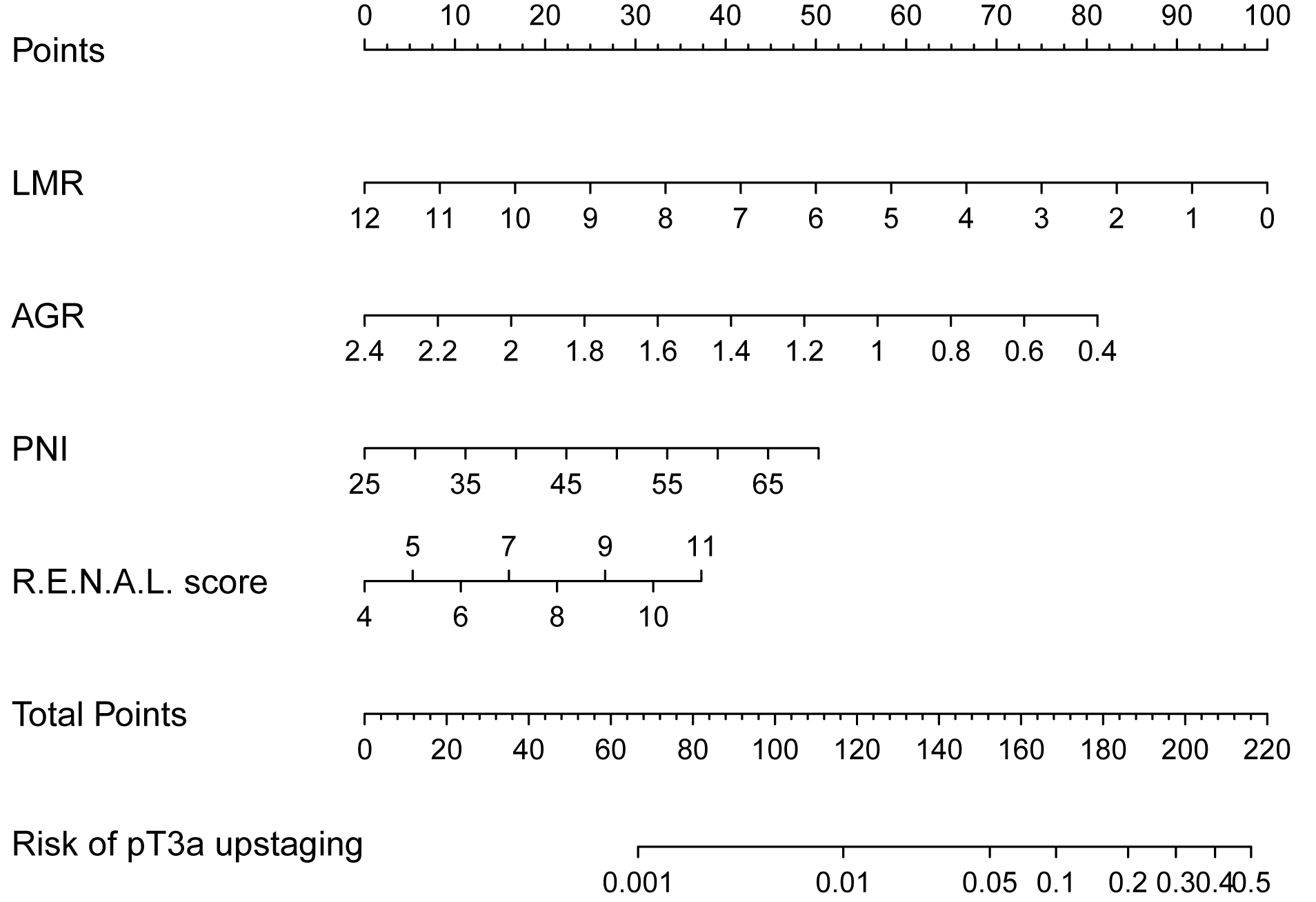

Supplement: Supplementary Figure 1 — Nomogram for predicting probability of pT3a upstaging for newly diagnosed cT1 RCC patients. [file Image_1.tif]
